# Function and regulation of ubiquitin-like SUMO system in heart

**DOI:** 10.3389/fcell.2023.1294717

**Published:** 2023-11-16

**Authors:** Ying Wang, Zhihao Liu, Xiyun Bian, Chenxu Zhao, Xin Zhang, Xiaozhi Liu, Nan Wang

**Affiliations:** ^1^ College of Biotechnology, Tianjin University of Science and Technology, Tianjin, China; ^2^ Central Laboratory, The Fifth Central Hospital of Tianjin, Tianjin, China; ^3^ First Teaching Hospital of Tianjin University of Traditional Chinese Medicine, National Clinical Research Center for Chinese Medicine Acupuncture and Moxibustion, Tianjin, China; ^4^ State Key Laboratory of Modern Chinese Medicine, Tianjin University of Traditional Chinese Medicine, Tianjin, China; ^5^ Tianjin Key Laboratory of Epigenetics for Organ Development in Preterm Infants, The Fifth Central Hospital of Tianjin, Tianjin, China

**Keywords:** SUMO, heart, protein quality control, Ca^2+^ cycle, cardiac metabolism

## Abstract

The small ubiquitin-related modifier (SUMOylation) system is a conserved, reversible, post-translational protein modification pathway covalently attached to the lysine residues of proteins in eukaryotic cells, and SUMOylation is catalyzed by SUMO-specific activating enzyme (E1), binding enzyme (E2) and ligase (E3). Sentrin-specific proteases (SENPs) can cleave the isopeptide bond of a SUMO conjugate and catalyze the deSUMOylation reaction. SUMOylation can regulate the activity of proteins in many important cellular processes, including transcriptional regulation, cell cycle progression, signal transduction, DNA damage repair and protein stability. Biological experiments *in vivo* and *in vitro* have confirmed the key role of the SUMO conjugation/deconjugation system in energy metabolism, Ca^2+^ cycle homeostasis and protein quality control in cardiomyocytes. In this review, we summarized the research progress of the SUMO conjugation/deconjugation system and SUMOylation-mediated cardiac actions based on related studies published in recent years, and highlighted the further research areas to clarify the role of the SUMO system in the heart by using emerging technologies.

## 1 Introduction

Protein post-translational modification (PTM) is crucial for regulating the conformational changes, activity and function of proteins, and plays a key role in life activities such as signal transduction, cell proliferation and differentiation, metabolism and tumorigenesis. PTM of protein has been shown to be closely related to cardiac dysfunction. PTM includes phosphorylation, acetylation, nitration, ubiquitination, as well as SUMOylation. The small ubiquitin-related modifier (SUMOylation) system is a reversible post-translational modification process, which can modulate the activity, location and stability of proteins by covalently binding to the substrate lysine residues to change their charge and modify the protein structure (Yau et al., 2021). The SUMOylation of proteins is catalyzed through the transfer of SUMO by SUMO-specific activating enzyme (E1) to the cysteine residue of the binding enzyme (E2), and the particular binding of SUMO to the substrate protein is finished with the formation of an isopeptide bond with lysine residues using ligase (E3) (Lascorz et al., 2022). The reverse reaction (i.e., lysine deSUMOylation) is catalyzed by sentrin-specific proteases (SENPs), which can cleave the isopeptide bond of a SUMO conjugate (Kerscher et al., 2006; van der Veen and Ploegh, 2012).

Studies on the intracellular SUMO modification have confirmed that SUMO modification can affect the function of proteins via various pathways (Li et al., 2021). Despite the recognition of its regulation of protein-protein interaction, recent studies have found that SUMO modification may also affect the interaction between chromatin protein and DNA (Li et al., 2020; Mitra et al., 2020). Additionally, SUMOylation has also been reported to modulate the activity of proteins in many vital cellular processes, including cellular communication (Oh et al., 2018), gene transcription (Ryu et al., 2019), metabolism (Castro et al., 2015) and apoptosis (Hu et al., 2021). Moreover, mammalian *in vivo* and *in vitro* studies have also manifested the intimate relationship between SUMOylation and numerous diseases, including neurological diseases (Henley et al., 2018), cancer (Seeler and Dejean, 2017; Kroonen and Vertegaal, 2021), and cardiovascular diseases (Vejpongsa and Yeh, 2014; Mendler et al., 2016). This suggests that a deeper understanding of the role of protein SUMOylation in these diseases can facilitate the pursuit of novel therapies.

Studies have connfirmed that SUMO conjugation/deconjugation system is closely related to heart development. Homozygous SUMO1 knockout mice exhibit high mortality due to both atrial septal defect (ASD) and ventricular septal defect (VSD), which is rescued by re-expressing of SUMO1 in the heart (Wang et al., 2011). SUMO2 deficiency also results in embryonic lethal, while SUMO3 deficiency is considered to be dispensable for heart development (Wang et al., 2014). In another study, mutated mice completely lacking SUMO1 can survive without any obvious phenotype, suggesting a compensatory effect of SUMO2/3 on SUMO1 deficiency (Evdokimov et al., 2008). Therefore, establishing and analyzing cardiac-specific conditional mice with either SUMO knockout/knockdown or overexpression is helpful to further understand the role of SUMO in the development of heart.

In the past decade, the number of studies on the modulation of SUMOs and SENPs in cardiovascular diseases (CVDs) has increased, and many SUMOylation-dependent regulatory mechanisms have been described in the context of cardiac hypertrophy (Gupta et al., 2016), heart failure (Kho et al., 2015) and myocardial ischemia reperfusion (I/R) injury (Chen et al., 2019). On the one hand, SUMO1 modification of SERCA2a has been reported to promote heart failure and induce cardiac hypertrophy (Buccarello et al., 2020). On the other hand, SUMO1 could improve myocardial fibrosis through PI3K-mediated SUMOylation of SERCA2a (Hu et al., 2017). SUMO also plays an important role in the treatment of cardiac I/R injury, because HIF1α SUMOylation mediated by SUMO1 is involved in the expression of vascular endothelial growth factor (VEGF) (Liu et al., 2021). SUMOylation modification has a dual role in heart diseases, but the specifics need to be further explored.

Several studies suggest that SUMO modification may play an important role in the development of cardiotoxicity and the treatment of cardiotoxic diseases. Doxorubicin (DOX) is a widely used chemotherapy drug in clinical practice, but could cause dose-dependent acute or chronic cardiotoxicity (Swain et al., 2003). DOX stimulation usually led to excessive accumulation of ROS and oxidative stress. H_2_O_2_ induced SUMOylation of ERK5 and inhibited ERK5 transcriptional activity, leading to apoptosis in cardiomyocytes (Shishido et al., 2008). Activation of ERK5 has been reported to inhibit cardiac apoptosis and dysfunction in Dox-treated mice (Yan et al., 2007). Dominant negative form of Ubc9 and a siRNA specific for PIAS1 resulted in the impair of ERK5-SUMOylation and the restoration of ERK5 transcriptional activity in cardiomyocytes (Shishido et al., 2008). Manish K Gupta explored the protective role of SUMOylation on cardiac proteotoxicity using transgenic mice overexpressing UBC9 specifically in cardiomyocytes. Cardiomyocyte-specific overexpression of UBC9 could improved cardiac function in in the mice with cardiac proteotoxic disease by increasing the level of SUMOylation and inducing autophagy (Gupta et al., 2016).

In this review, we focus on the SUMO conjugation/deconjugation system, and the role of SUMO system components in regulating cardiac metabolism, Ca^2+^ cycle, ion channels and cardioprotein toxicity. We also discuss the action potential of the SUMO/deSUMO system in cardiac mitochondrial and endoplasmic reticulum and its physiological and pathological relevance. Finally, we envision how novel findings and technologies can enable breakthroughs in heart research, ultimately leading to innovative treatment strategies for cardiovascular diseases.

## 2 SUMO conjugation/deconjugation system

Four SUMO isoforms (SUMO-1, -2, -3, -4) have been identified in mammals, which are characterized by an evolutionary conserved ubiquitin fold-like structure (Chang and Yeh, 2020). SUMO-1 contains 101 amino acids, including a flexible NH2 terminus, a ubiquitin fold (amino acids 22-97), and a short COOH terminus. SUMO-2 and -3 contain 103 and 95 amino acids, respectively. However, in organisms, SUMO-2 and -3 (also known as SUMO-2/3) share ≈95% sequence identity, but with low sequence similarity with SUMO1 (≈50%) (Hay, 2005). SUMO-4 shares very similar sequence identity with SUMO-2/3, but contains proline instead of glutamine at position 90, which may prevent SUMO-4 from being processed and conjugated under normal conditions (Owerbach et al., 2005). Nevertheless, the observation of mature SUMO-4 in lysates derived from serum-starved cells suggests the use of SUMO-4 in response to protein-starvation under stress conditions (Wei et al., 2008).

All SUMO genes actually encode a SUMO precursor protein with a short C-terminal peptide, which is cleaved and degraded by proteases (SENPs in human and ULP1 in yeast) to produce mature SUMO with diglycine residues (Hickey et al., 2012). Similar to the ubiquitination cascade, the SUMO conjugation reaction involves three steps. Specifically, SUMO activating enzyme (E1, an ATP-dependent heterodimer made up of SUMO-activating enzyme subunit 1 (SAE1, also known as Aos1) and SUMO-activating enzyme subunit 2 (SAE2, also known as Uba2) binds to the C-terminal amino acid of mature SUMO with diglycine residues. In the activation stage, ATP is hydrolyzed to produce a high-energy SUMO-E1 thioester bond, allowing the SUMO-E1 complex to assemble. UBC9 can be recognized precisely by SAE1/SAE2 (the only E2 binding enzyme currently known). By the transesterification event, SUMO is transferred to the cysteine residue of UBC9 and produces an SUMO-E2 thiolester intermediate. Finally, the conserved tetrapeptide motif ψ-KxD/E (ψ-hydrophobic residues, K-lysine, x-any amino acid, and D/E-acidic residues) is recognized by UBC9 through catalysis of E3 ligase, enabling the binding of SUMO to lysine residues to form an isopeptide bond and complete the specific binding of SUMO to the substrate protein. E3 ligase is generally believed to have the ability to enhance the recognition between UBC9 and the substrate and augment the efficiency of SUMO conjugation to the substrate. SUMO E3 ligase was initially identified to hold a conserved RING-finger domain, but at least two family members were shown to not require this domain for their ability to mediate substrate SUMOylation (Pichler et al., 2002; Ivanov et al., 2007). Subsequently, the SUMO E3 ligase was considered able to bind to UBC9 and SUMO, and could enhance the transfer efficiency/capability of SUMO molecules from E2 enzyme substrate protein, thus expanding the SUMO E3 enzyme family members. A variety of E3 ligases have been identified, including the family of protein inhibitors of activated STATs (PIAS) (PIAS1, PIAS2, PIAS3, PIAS4) (Li et al., 2023), polycomb protein 2 (Pc2) (Roscic et al., 2006), nonstructural maintenance of chromosomes element 2 (Mms21) (Zhao and Blobel, 2005), ras-related nuclear protein binding protein 2 (RanBP2) (Pichler et al., 2002), TOP1 binding arginine/serine rich protein (TOPORS) (Pungaliya et al., 2007), mitochondrial-anchored protein ligase (MAPL) (Braschi et al., 2009), zinc finger protein associated with TDP2 and TOP2 (ZATT) (Tian et al., 2021), an tripartite motif-containing protein 28 (TRIM28) (Li et al., 2020). SUMO-2 and -3 contain the ψ-KxE sequence in their N-terminal extension, which can be used as the SUMO connection site to form a poly-SUMO chain, while SUMO1 cannot form a chain-like combination due to lack of this motif (Jansen and Vertegaal, 2021). Note that there is only one E1 enzyme and E2 enzyme known for SUMO conjugation, in contrast to the involvement of a large number of E1 and E2 enzymes in the ubiquitination cascade, and some specific substrates can be SUMOylated with E1 and high concentrations of UBC9 *in vitro* (Coey et al., 2014). However, how the organism can complete the specific SUMO modification of a large number of substrates only through SAE1/SAE2 and UBC9 remains a mystery.

**FIGURE 1:**
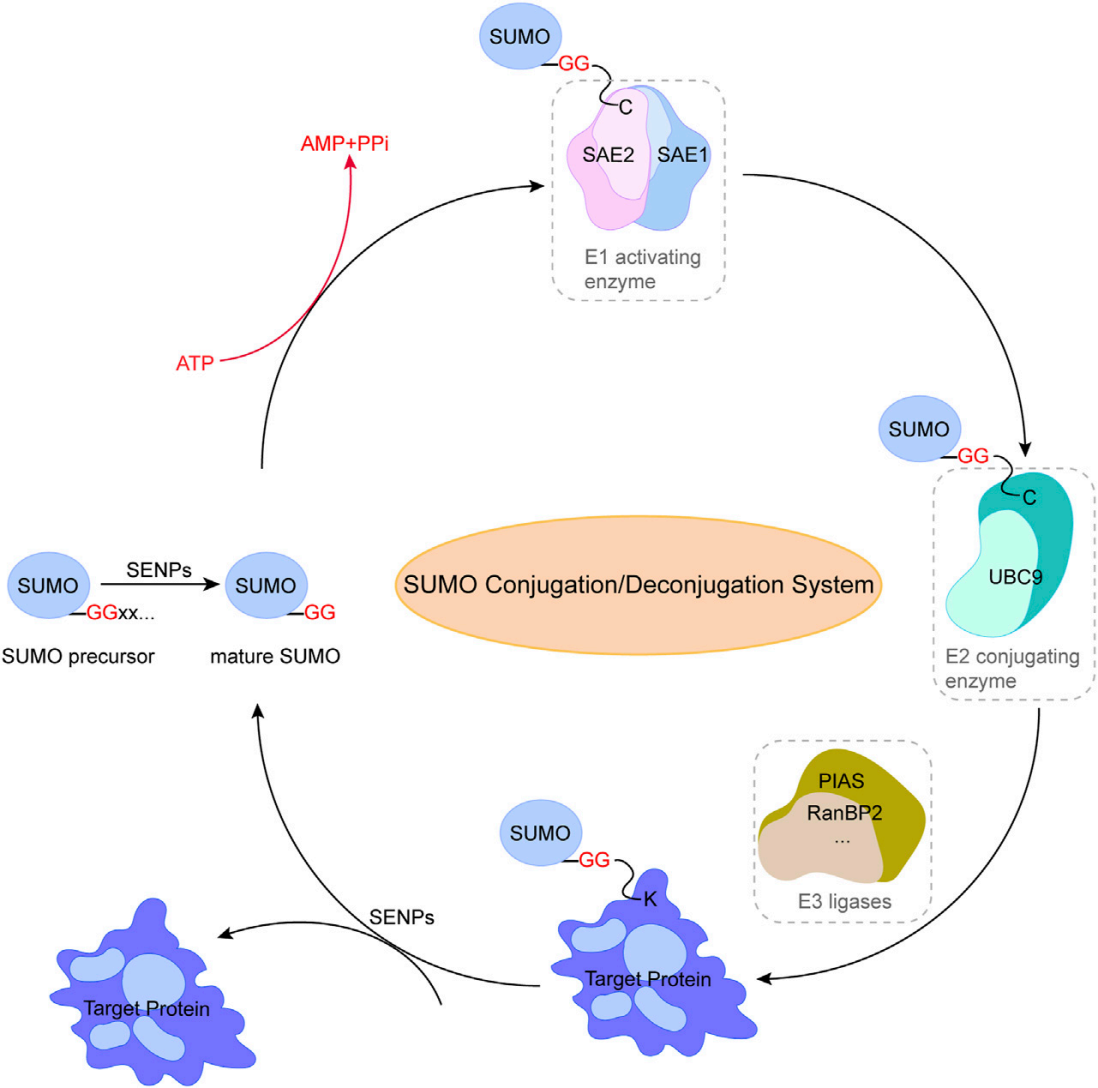
Pathway of small ubiquitin-related modifier (SUMO) conjugation/deconjugation system. SUMO precursor is cleaved and degraded by SENPs to produce mature SUMO with diglycine residues. SUMOylation of proteins is catalyzed by SUMO-specific activating enzyme (E1) through the transfer of SUMO to the cysteine residue of binding enzyme (E2), followed by the binding of SUMO to lysine residues through ligase (E3) to form an isopeptide bond and complete the specific binding of SUMO to the substrate protein. The reverse reaction is catalyzed by SENPs. This figure was created using Adobe illustrator cc 2018 (https://www.adobe.com/cn/products/illustrator.html).

SUMO conjugations are a form of reversible modification to break the isopeptide bond by hydrolases specific to SUMO. The primary family of SUMO-specific hydrolases/proteases in human cells is a family of isopeptidases known as SENPs (Chang and Yeh, 2020). Two additional hydrolases families have been identified, including the deSUMOylation hydrolases of deSUMOylating isopeptidase1 (DeSI1) (Suh et al., 2012) and ubiquitin-specific protease-like 1(USPL1) (Schulz et al., 2012), but their specificity and function for SUMO-modified substrates have not been clarified. SENPs are essential to maintain the balance of the SUMO coupling/uncoupling system due to their dual function in both maturation and uncoupling of SUMO (Figure 1), and to date, six members of the SENPs family with the ability of deSUMOylation have been identified (SENP-1, -2, -3, -5, -6, -7). In addition, SENPs display deconjugation specificity to SUMO family members. Specifically, SENP1 and SENP2 actually target all SUMO isoforms for deconjugation (García-Gutiérrez and García-Domínguez, 2022), SENP3 and SENP5 mainly recognize SUMO2/3 and remove them from the substrate, and SENP6 and SENP7 can ablate the polymeric chains of SUMO2/3, thereby breaking the connection between the polymer chain and the target protein (Vertegaal, 2022). In general, SUMOylation and deSUMOylation together constitute a complete reversible enzyme reaction chain, further regulating various cellular biological processes through SUMO and substrate conjugation and deconjugation.

The data from patients and animal models have shown that the balance between SUMO binding and unbinding is a key issue in cardiac stress adaptation. In a pressure overload-induced model of myocardial hypertrophy and heart failure, elevated SUMO1 conjugation was shown to suppress the hypertrophy phenotype and inhibit the hypertrophic responses in cultivated cardiomyocytes (Hotz et al., 2021). Additionally, emerging research proves that the SUMO system is related to the occurrence and development of human heart diseases (Xiao et al., 2022).

## 3 SUMO and protein quality control in the heart

To maintain the homeostasis of protein environment, cardiomyocytes rely on a signaling network of factors and pathways collectively known as protein quality control (PQC) system, which can not only mediate the accurate folding and assembly of initially synthesized proteins, but can also monitor the refolding and degradation of misfolded proteins (Wang and Hill, 2015; Sciarretta et al., 2018). There are two major pathways for the PQC system to remove misfolded proteins, including the ubiquitin–proteasome system (UPS) and autophagy, which can lessen the buildup of protein toxicity in cardiomyocytes by eliminating improperly folded proteins.

Initially, SUMO modification was speculated to antagonize ubiquitination and maintain protein stability due to the competitive binding of SUMO molecules and ubiquitin molecules to the same lysine site of the substrate protein. However, subsequent research has discovered that SUMO can synergize the ubiquitin degradation effect by promoting multiple ubiquitination (Liu et al., 2021), and the degradation of misfolded proteins and aggregates in cardiomyocytes involves the SUMO cascade system (Ahner et al., 2013; O'Rourke et al., 2013). UBC9, as the only E2 enzyme in the SUMO cascade, plays an essential role in the PQC system of cardiomyocytes. UBC9 knockdown *in vivo* could trigger a significant accumulation of aggregated proteins by inhibiting the ability of UPS-mediated protein degradation, leading to protein toxicity in cardiomyocytes, while UBC9 overexpression could significantly decrease the harmful preamyloid oligomers and misfolded proteins in the context of protein toxicity (Gupta et al., 2014). Promyelocytic leukemia protein (PML) covalently modified by SUMO is degraded in a ubiquitin-mediated manner through the SUMO-dependent ubiquitin E3 ligase family member RNF4. Restraint of SUMOylated PML by silencing UBC9 was shown to attenuate cardiac fibrosis and partially improve cardiac function in transverse aortic constriction (TAC) mice, while enhancing SUMOylated PML by silencing RNF4 was found to attenuate the accumulation of PML *in vivo* (Dai et al., 2020). However, no report is available on the influence of SUMO and ubiquitin system in regulating the abundance of PML on PQC system.

SUMO was also shown to have regulatory effects on autophagy, another pathway of the PQC system. Autophagy is a process where misfolded or damaged cellular proteins, organelles or protein aggregates are encapsulated in a double-membrane structure and degraded by lysosomes (Saito and Sadoshima, 2015; Delbridge et al., 2017). Both UBC9 overexpression in cardiomyocytes (Gupta et al., 2016) and myocardial injection of adenovirus carrying UBC9 (Xiao et al., 2020) were shown to increase autophagy flux in mice with cardiac proteotoxicity, enabling the mice to improve intracellular protein environment homeostasis and cardiac contractile function, indicating the necessity of the SUMO system for effective autophagy and cardiac function. UBC9, as the only E2 enzyme in the SUMO cascade, has a profound impact on global SUMO conjugation in the heart (not only regulating the UPS system, but also autophagy). Understanding the link between SUMO and the PQC system may yield new targets for treatment of its associated cardiac diseases.

## 4 SUMO and Ca^2+^ cycle in the heart

**FIGURE 2:**
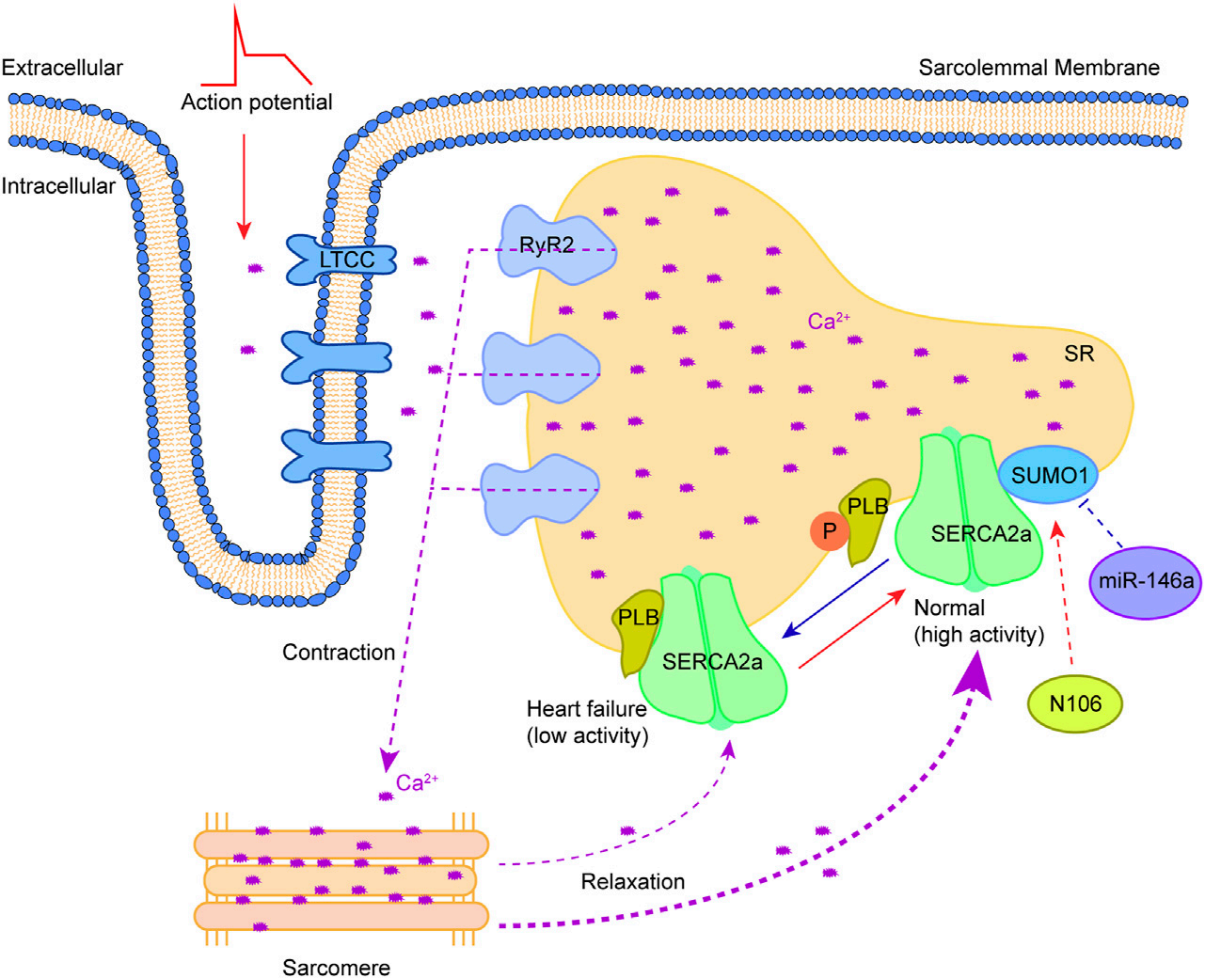
Therapeutic approaches to target SUMOylation of SERCA2a. Myocardial cell membrane depolarization triggers the entry of extracellular Ca^2+^ into the cytosol through LTCC. RyR2 activation on SR results in releases of a large amount of Ca^2+^ to the cytosol (Ca^2+^-induced Ca^2+^ release), consequently causing the binding of Ca^2+^ to troponin C to trigger contraction. Relaxation occurs after Ca^2+^ removal in the cytosol by SERCA2a. The SERCA2a activity is regulated by PLB along with a combination of SUMO1 modification. Phosphorylation of PLB and SUMOylation by SUMO1 increase it. Reversing the abnormality of SERCA2a SUMOylation in heart failure by drug (N106) or microRNA (miR-146a) holds promise for heart failure treatment. This figure was created using Adobe illustrator cc 2018 (https://www.adobe.com/cn/products/illustrator.html).

Ca^2+^, an important second messenger in cells, is widely involved in regulating cell proliferation, apoptosis, differentiation and other important physiological processes. In cardiomyocytes, the concentration changes of extracellular and intracellular free Ca^2+^ play a critical role in heart contraction and relaxation (Tilemann et al., 2013; Dridi et al., 2020). In the time of myocardial cell membrane depolarization, extracellular Ca^2+^ enters the cytosol through the L-type Ca^2+^ channel (LTCC) on the T-tubule, and the activation of the ryanodine receptor 2 (RyR2) on the sarcoplasmic reticulum (SR) results in the release of large amounts of Ca^2+^ to the cytosol (Ca^2+^-induced Ca^2+^ release), followed by the binding of Ca^2+^ to troponin C to promote coarse/thin filament slip when the cytosolic Ca^2+^ concentration rises to a critical level (Houser, 2014). Sarcoplasmic/endoplasmic reticulum Ca^2+^ ATPase 2a (SERCA2a) is responsible for removing Ca^2+^ in the cytoplasm. Phospholamban (PLB) regulates the ability to recapture Ca^2+^ of SERCA2a. The conversion of PLB monomers to multimers could enhance SERCA2a activity, leading to the recovery of sufficient Ca^2+^ into SR. Studies in genetically altered animal models have defined the functional role of the SERCA2a pump in Ca^2+^ homeostasis and cardiac physiology (He et al., 2020; Zhihao et al., 2020). The weakening of SERCA2a function in failing hearts was shown to decrease the Ca^2+^ content of SR, leading to systolic dysfunction. Meanwhile, the diminished SERCA2a activity could also reduce the amount and rate of Ca^2+^ removal from cytoplasm, contributing to diastolic dysfunction (Gorski et al., 2019). In the study of Kho et al. (Kho et al., 2011), the SERCA2a ATPase activity and stability was shown to be regulated by SUMO1, and this activity and stability, as well as its ability to take up intracytoplasmic Ca^2+^, presented a positive effect when SERCA2a was largely SUMOylated. Importantly, the SUMOylation of SERCA2a could enhance the cardiac function of mice with concomitantly impaired Ca^2+^ homeostasis and heart failure. Mutation of lysine at positions 480 and 585 to arginine could reduce the SUMOylation level of SERCA2a and the ATPase activity (Figure 2). Increasing the levels of SERCA2a SUMOylation could improve myocardial systolic-diastolic function and reduce cardiomyocyte death (Peng et al., 2023). SUMO1 is a target gene of miR-146a and its expression level is negatively regulated by miR-146a (Kho et al., 2011). The high expression of miR-146a could cause a decrease in SERCA2a SUMOylation, Ca^2+^ transient impairment and contractility in heart failure. It is worth noting that miR-146a could reduce the level of SUMO1 *in vivo*, consequently leading to a significant effect on the level of SUMO1 substrates. In addition, the use of AAV-1 mediated SUMO1 gene transfer alone in the porcine model of chronic heart failure was also shown to enhance the mRNA level of SERCA2a and increase the distribution of SERCA2a in the SR of cardiomyocytes (Tilemann et al., 2013). Therefore, SUMO1 not only participates in the post-translational modification regulation of SERCA2a, but also regulates the expression level of SERCA2a *in vivo* by acting with its transcription factor SP1. The expression of SERCA2a after myocardial ischemia/reperfusion (I/R) injury was significantly reduced when transfected with SP1 knockdown adenovirus, suggesting that SP1 can promote SERCA2a expression at the transcriptional level (Hu et al., 2020). The binding of SUMO1 to SP1 was also found to enhance SERCA2a transcription, improve DNA binding and increase SP1 stability. Upregulating the expression of SUMO1 could increase the SUMOylation modification of SP1, thus promoting the transcription of SERCA2a (Hu et al., 2017). These results indicate that the regulatory role of SUMO1 is extremely extensive. However, the SUMO1-mediated SP1 SUMOylation usually results in the degradation of SP1 in a ubiquitin-dependent manner (Spengler and Brattain, 2006; Nolfi-Donegan et al., 2020). Therefore, the increase of SERCA2a activity *in vivo* triggered by SUMO1 overexpression may be due to the direct enhancement of SERCA2a stability (Kho et al., 2011). The small molecule N106 (N-(4-methoxybenzo [d]thiazol-2-yl)-5-(4-methoxyphenyl)-1,3,4-oxadiazol-2-amine) could augment the SUMOylation level and SERCA2a activity by directly targeting the SUMO E1 enzyme, thus enhancing the contractility and calcium transients of adult cardiomyocytes in a dose-dependent manner (Kho et al., 2015). The pocket between subunit 1 (SAE1) and subunit 2 (SAE2) of the SUMO E1 enzyme could accommodate N106, allowing the establishment of the N106-SAE1 complex by connecting with position 315 of valine and position 312 of glutamine on SAE1 and the amelioration of its enzyme activity. It is worth noting that N106 could increase SERCA2a SUMOylation via substrates, although it targets the SUMO E1 enzyme. The change degree in SUMOylation of individual proteins varies with N106 treatment. This can be attributed to the wide range of SUMO substrates and the scarcity of SUMO E1 enzymes (currently only one is found, namely, the SAE1/SAE2 complex), which may restrict the development and application of N106, due to its involvement in the regulation of other substrates, but these effects are unknown and need to be further elucidated.

Cardiac troponin is a key regulatory protein that can control cardiac muscle contraction and relaxation in a calcium-dependent manner. It is composed of three subunits, including the calcium-binding subunit troponin C (TnC), the inhibitory subunit troponin I (TnI), and the tropomyosin-binding subunit troponin T (TnT). TnI is unique to myocardial cells, and an increase in TnI content usually leads to several cardiac diseases such as myocardial infarction and myocarditis. TnI has been recently reported to be SUMOylated at lysine 177. SUMOylation of TnI at K177 does not affect its stability, but prevents other myofilament proteins nearby from being SUMOylated. Furthermore, overexpression of K177R mutants can reverse this blockade of SUMOylation, allowing SUMO conjugation of other proteins (Fertig et al., 2022).

## 5 SUMO and ion channel in the heart

SUMO conjugation/deconjugation can also regulate cardiac ion channel activity, which is related with arrhythmia. Mutations in inward rectifying potassium channel (IK1) function are commonly associated with atrial fibrillation, supraventricular tachycardia, and Andersen Tawil syndrome (also known as type 7 long QT syndrome), among others. IK1 is primarily delivered by inward rectifying K+ channel (Kir2.1), and Kir2.1 maintains its activity through the membrane phospholipid phosphatidylinositol 4,5-bisphosphate (PIP2). Acute hypoxia usually leads to rapid SUMOylation of Kir2.1 channels in cardiomyocytes, but SUMO1 can inhibit IK1 by reducing PIP2-mediated activation of Kir2.1 channels (Xu et al., 2022). The abnormal function of cardiac slow delayed rectifier K+ current (IKs) channel can cause long QT syndrome (QT interval prolongation) and life-threatening arrhythmia (Bohnen et al., 2017). The IKs channel is composed of four KCNQ1 pore-forming subunits and two KCNE1 accessory subunits. SUMO2 binds to each KCNQ1 subunit at position 424 of lysine, which can shift the half-maximum activation voltage of IKs to ∼+8 mV (Xiong et al., 2017). IKs channels could be SUMOylated 4 times, thereby producing a right-shift of ∼34 mV in neonatal mouse cardiomyocytes or Chinese hamster ovary cells. In the mammalian heart, the Nav1.5 voltage-gated sodium channels could maintain the cardiac action potential platform through the late sodium current (ILATE) (Makielski and Kyle, 2015). Acute cardiac hypoxia/ischemia can lead to excessive ILATE, thus prolonging action potential duration (APD), decreasing repolarization reserve and increasing susceptibility to after-depolarizations, which are common factors of dangerous arrhythmia. In the study by Plant et al. (Plant et al., 2020), acute cardiac hypoxia was reported to increase ILATE and cause arrhythmia, which is caused by the rapid SUMOylation (Lysine 442 site) of the Nav1.5 channel located in the cell membrane. Adding SUMO1 could increase ILATE to ∼4.2% of peak INa (essentially for maintaining APD rise) just like the case of hypoxia. Consistent with the effect of K442 mutation at the SUMOylation site of Nav1.5 and the inhibitor of ILATE, SENP1 could reduce ILATE in human cardiomyocytes derived from pluripotent stem cells. Moreover, hyper-SUMOylation of the Kv7 potassium channel could cause spontaneous seizures and sudden death in SENP2-deficient mice (Chen et al., 2021). These mice showed atrioventricular conduction blocks and cardiac asystole, indicating the requirement of functional copy of the SENP2 gene for effective excitability and electrical conduction of cardiomyocytes.

## 6 SUMO and mitochondrial fission in the heart

Substrates modified by SUMO are not limited to the nucleus, and several important targets are localized in the cytoplasm and contact other intracellular organelles. Substantial SUMO1 conjugates were observed in mitochondrial fractions, indicating the occurrence of extensive SUMO modification in mitochondria (Zunino et al., 2007). Mitochondrial fission is necessary to maintain the mitochondrial network by relying on a large GTPase called dynamin-related protein 1 (DRP1, also known as DNM1L). Under normal conditions, Drp1 is primarily located in the cytosol. During mitochondrial fission, Drp1 is recruited to the surface of the OMM by four OMM receptors and/or adapters including fission 1 (Fis1), mitochondrial fission factor (Mff), and mitochondrial dynamics protein 49 (MiD49) and MiD51. It assembles around the mitochondria in a helical oligomer and results in the constriction and scission of the organelle in a GTP-dependent manner. At the end of the division, Drp1 returns to the cytosol. Mitochondrial-anchored protein ligase (MAPL), as the E3 ligase of SUMO, positively regulates mitochondrial fission by attaching SUMO to DRP1 (Braschi et al., 2009; Mohanty et al., 2021). In a previous study (Zhao et al., 2017), both increased MAPL expression and elevated mitochondrial fission were observed in mice with phenylephrine (PE)-induced cardiac hypertrophy. MiR-485-5p affected SUMO1 modification of DRP1 and negatively regulated mitochondrial fission by inhibiting the expression of MAPL. In addition, augmenter of liver regeneration (ALR) (also known as hepatic stimulatory substance or hepatopoietin) was found to prevent mitochondrial fission by inhibiting DRP1 SUMOylation, thus rescuing hepatocytes from IR-induced apoptosis (Huang et al., 2021).

DRP1 is not only a substrate of SUMO1, but also can be modified by SUMO2/3, and the regulation of DRP1 by different SUMO molecules is inconsistent. In contrast to the reported pro-apoptotic effects of SUMO1 conjugation, the SUMO2/3 modification of DRP1 was shown to have the cytoprotective effect in an *in vitro* model of ischemia (Huang et al., 2021). SUMOylation can also affect the localization of Drp1. SUMO1 modification of Drp1 promotes its localization in mitochondria, while SUMO2/3 conjugation inhibits its binding to the mitochondrial membrane. Drp1 SUMOylation induced by Zinc increased mitophagy during reperfusion, thereby inhibiting ROS production and myocardial injury (Bian et al., 2019). SENP2 has been considered as a key protease for removing SUMO1 modification of Drp1 (Bian et al., 2019). The depletion of SUMO-2/3-specific protease SENP3 could stabilize SUMO modification level of DRP1, thus reducing DRP1-mediated cytochrome c release and caspase-mediated cell death (Guo et al., 2013; Guo et al., 2017). Post-translational modifications of Drp1 regulate its activity, enabling it to adapt to different stimuli, but also exposing it to danger. DRP1 and its specific SENP may exert different functions depending on the state of cells and animal models. Therefore, it is particularly important to regulate DRP1 SUMOylation by targeting different SENPs.

## 7 SUMO and metabolism in the heart

**FIGURE 3:**
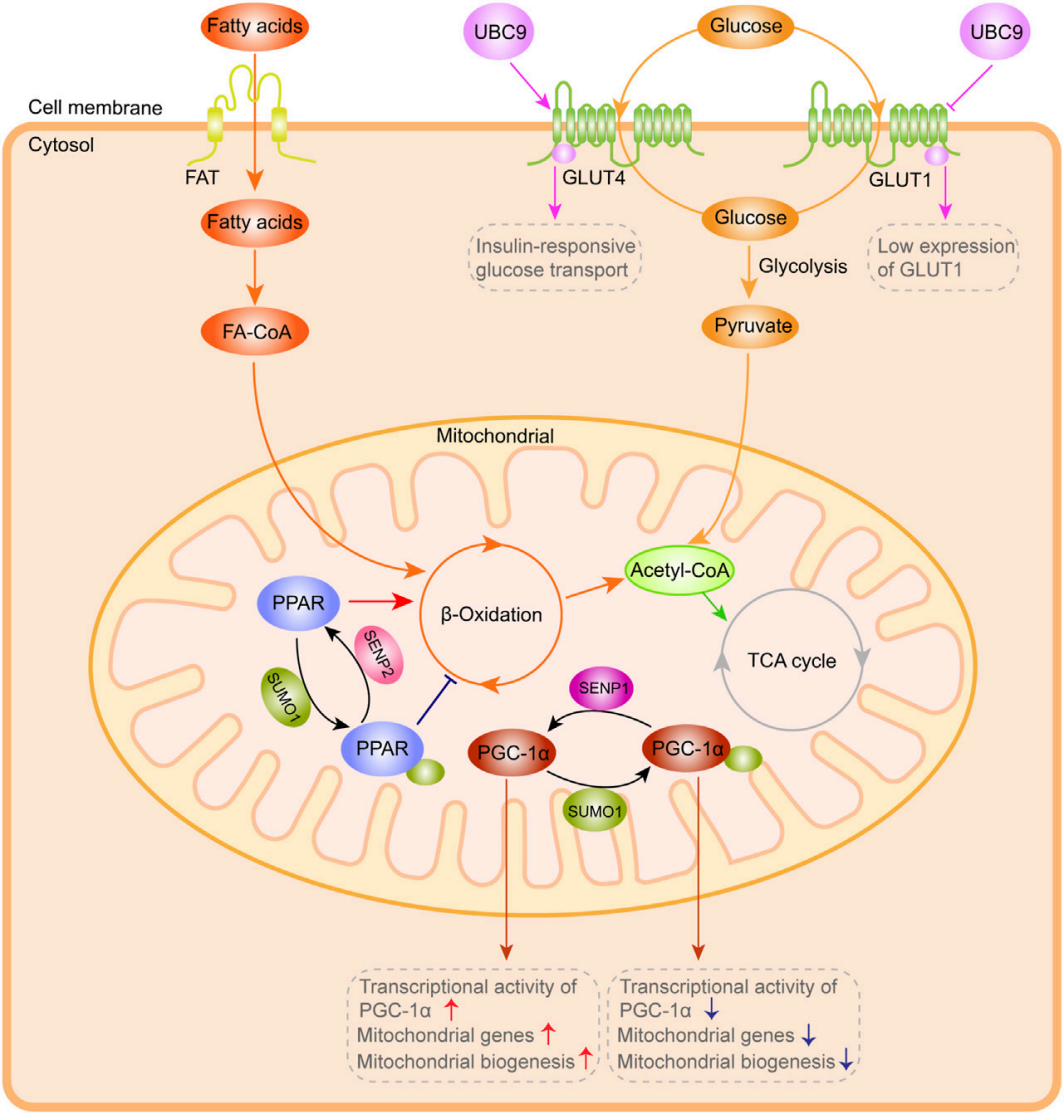
SUMO system-mediated action in cardiac metabolism. Exogenous and glycogen-derived glucose is transported to the cytoplasm by GLUT1 and GLUT4. UBC9 overexpression enhances GLUT4 transporter abundance and interacts with GLUT4, leading to a significant augment in the insulin-responsive glucose transport, coupled with low GLUT1 expression and decreased basal glucose transport. PPAR and PGC-1α are highly dynamically controlled by the SUMO system, which can affect *ß*-Oxidation efficiency, PGC-1α transcriptional activity and mitochondrial biogenesis. This figure was created using Adobe illustrator cc 2018 (https://www.adobe.com/cn/products/illustrator.html).

Maintaining the effective pumping capacity, metabolic process and ion homeostasis of the heart requires a constant energy supply. The heart provides ATP primarily through mitochondrial oxidative phosphorylation, and cardiomyocytes rely on mitochondria to provide more than 95% of the energy required for their function (Nolfi-Donegan et al., 2020). Cardiomyocytes undergo metabolic remodeling (conversion of substrate utilization from fatty acids to glucose) in hypertrophic hearts or during heart failure (Bertero and Maack, 2018; Birkenfeld et al., 2019). The activity of the rate-limiting enzyme and the number of available enzyme molecules, which can be regulated by post-translational modifications, jointly determine the flow of substrate into the oxidation pathway in the cardiomyocytes. In recent studies, SUMO has been shown to mediate the cardiac metabolism by regulating transcription and post-transcription in the process of heart hypertrophy and failure (Figure 3).

Exogenous and glycogen-derived glucose is transported to the cytoplasm by glucose transporters (including GLUT1 and GLUT4), and then rapidly phosphorylated to glucose-6-phosphate under the catalysis of hexokinase in cardiomyocytes (Tian and Abel, 2001; von Lewinski et al., 2010). In previous studies (Lalioti et al., 2002), UBC9 was determined as a protein that interacts with GLUT4 and GLUT1 glucose transporters. Overexpression of Ubc9 could upregulate GLUT4 abundance and thus augment the insulin-responsive glucose transport, while simultaneously downregulating GLUT1 abundance and reducing the basal glucose transport. In the study of Liu et al. (2007), overexpression of Ubc9 was shown to reduce GLUT4 degradation and promote its targeting to the distinct insulin-responsive GLUT4 storage compartment, resulting in an augment in the amount of GLUT4 and insulin-responsive glucose transport in adipocytes.

In the heart, 50%-70% of energy (in the form of ATP generation) is derived from *ß*-oxidation of fatty acids (FA), and the enzymes involved in FA oxidation are highly transcriptionally controlled. The most widely studied is the peroxisome proliferator-activated receptor (PPAR) family, including PPARα, PPARβ and PPARγ, with the former two as the main subtypes expressed in the heart, which extensively regulate mitochondrial biogenesis and substrate oxidation (Montaigne et al., 2021). PPAR activated by the ligands (lipid metabolites) could form a heterodimer with the retinoid X receptor, which binds to the PPAR response element (PPRE) in the promoter region of the target genes to regulate their expression. Overexpression of PPARα in the heart could trigger a significant increase in FA uptake and oxidation, due to the high regulation of the enzymes involved in FA oxidation by PPARα (Banke et al., 2010; Planavila et al., 2011). Apart from binding to ligands, PPAR can be regulated by SUMO. Lysine 185 in the hinge region of PPARα can bind to SUMO1, which is mediated by Ubc9 and SUMO E3-ligase PIASy (Pourcet et al., 2010). PIASy is an important target in diseases such as ischemic heart disease and life-threatening arrhythmias. I/R-induced activation of cardiac PIASy could increase SUMOylation of caveolin-3 (Cav-3) by SUMO2/3, leading to ventricular arrhythmias (Hu et al., 2022). Although the expression of PPARγ is limited in the heart, the ligand-dependent SUMOylation of PPARγ allows its recruitment to the promoters of inflammatory genes to participate in the regulation of these genes (Zelcer and Tontonoz, 2005). In addition, SENP2 overexpression could substantially enhance FA oxidation in myotubes, and ultimately alleviate obesity and insulin resistance caused by high-fat diets (Koo et al., 2015; Koo et al., 2019), suggesting the importance of the balance of SUMOylation and deSUMOylation in diabetic heart disease.

Peroxisome proliferator-activated receptor γ coactivator-1α (PGC-1α), a tissue-specific transcriptional coactivator closely related to energy metabolism, can enhance the activity of many nuclear receptors. The expression of PGC-1α is sufficient to maintain the increase in fatty acid oxidation rate in the heart after birth. In cultured cardiomyocytes, the upregulation of PGC-1α could increase the number of mitochondria and the gene abundance of related metabolic enzymes, accompanied by enhanced FA oxidation and coupled respiration rate (Guo et al., 2014). In addition to PPARγ, PGC-1 can bind with other several nuclear receptors, such as nuclear respiratory factors (NRFs) and estrogen-related receptor α (ERRα) and trans-activate their target genes. PGC-1α activates the transcription of mitochondrial transcription factor A (TFAM), mitochondrial transcription factors B1 and B2 (TFB1M, and TFB2M) by binding with NRF-1 and NRF-2, and these mitochondrial specific transcription factors further regulate the transcription of multiple genes required for mitochondrial respiration, including *ß*-ATP synthase, COX II, COX IV, cytochrome c and so on (Wu et al., 1999). Furthermore, PGC-1a is found to act as a coactivator of p53 and enhance its transcriptional ability, therefore resulting in augmenting cell-cycle arrest and promoting cell survival in response to metabolic stress (Sen et al., 2011). Interestingly, the SUMO1 conjugation of conserved lysine residue 183 located in the activation domain of PGC-1α can enhance the interaction of PGC-1α with corepressors to inhibit its transcription (Rytinki et al., 2009). Another study confirmed that SUMOylation at lysine 183 can enhance the interaction between PGC-1α and RIP40, thus reducing the transcriptional activity of PGC-1α (Rytinki and Palvimo, 2009). The deSUMOylation ability of SENP1 can enhance the transcriptional activity of PGC-1α, which is vital for the expression of mitochondrial genes and subsequent mitochondrial biogenesis (Taghvaei et al., 2022).

Adenosine monophosphate-activated protein kinase (AMPK) acts as an energy sensor and is activated to adjust multiple metabolic pathways in response to lowered energy levels. AMPK maintains the energy homeostasis of cardiomyocytes and plays an important role in regulating cardiomyocyte metabolism. The AMPK signaling pathway is closely related to cardiomyocyte survival and AMPK activation is found to protect the heart from apoptosis in response to various stimuli, including I/R, oxidative stress, etc (Bairwa et al., 2016). AMPK is known to inhibit the activity of mTORC1 signaling via promoting the phosphorylation of tuberous sclerosis 2 (TSC2) on S1345 or inhibiting the phosphorylation of the regulatory-associated protein of mTOR (Raptor) on Ser722 and Ser792 (Inoki et al., 2003). Yan et al. reported a new regulatory mechanism by which AMPK inhibits mTOR activity. The SUMOylation of AMPKα1 catalyzed by PIAS4 inhibits AMPK activity and further specifically regulates mTORC1 signalling (Yan et al., 2015). Interestingly, AMPK can be modified by SUMO2 instead of SUMO1, and this modification enhances AMPK activity (Rubio et al., 2013). Liver kinase B1 (LKB1) is an upstream kinase of AMPK. Overexpression of LKB1 can promote the activation of AMPK in cardiomyocytes, thereby inhibiting the growth of cardiomyocytes. Conversely, knockout of LKB1 can impair AMPK activation and induce cardiomyocyte hypertrophy (Ikeda et al., 2009). Interestingly, energy depletion caused the SUMOylation of LKB1 at lysine 178 and this modification promotes the interaction of LKB1 and AMPK, leading to the activation of AMPK signalling.

In summary, the SUMO system involves multiple links of energy metabolism, which are important for comprehending the balance of SUMOylation/deSUMOylation and metabolic homeostasis.

## 8 SUMO and endoplasmic reticulum stress in the heart

The endoplasmic reticulum (ER) is a multifunctional intracellular organelle with diverse functions, including protein folding, calcium homeostasis and lipid biosynthesis. The accumulation of unfolded proteins (known as ER stress) could be induced by stimuli such as ischemic injury (Feng et al., 2017), disturbance of Ca^2+^ homeostasis (Li et al., 2021), oxidative stress (Jin et al., 2017), and increased expression of normal and/or folding-defective proteins (Oakes and Papa, 2015). Abnormal ER stress responses can affect heart function, including disordered intracellular metabolism, abnormal cardiac development, myocardial I/R injury, cardiomyopathies and heart failure (Ren et al., 2021).

ER and mitochondria establish a dynamic interplay in regulating local Ca^2+^ communication and maintaining Ca^2+^ homeostasis. The physical contact sites between ER and mitochondria are called as mitochondrial associated membranes (MAMs), which contain multiple proteins involved in regulating calcium transport. The ER or sarcoplasmic reticulum (SR) are well known to be the main intracellular calcium ion pool, while mitochondria usually do not store calcium ions under physiological conditions. In response to various stimuli or stress, Ca^2+^ in the ER or SR is released into cytoplasm through inositol 1,4,5-triphosphate receptors (IR3Rs) or ryanodine receptors (RyRs) (Csordás et al., 2018). Then these calcium ions bypass the outer mitochondrial membrane (OMM) and enter the mitochondrial intermembrane space, which is mainly mediated by voltage-dependent anion channels (VDACs). IP3R1 and its companion proteins GRP75, as well as VDAC1, form the IP3R1-GRP75-VDAC1 complex, which is an important component of MAMs and participates in the delivery of Ca^2+^ from the ER to the mitochondrial intermembrane space. Mitochondrial matrix Ca^2+^ uptake is mediated by the mitochondrial calcium uniporter (mtCU) located in the inner mitochondrial membrane (IMM). During the early stages of ER stress, increased ER-mitochondrial connectivity could promote ATP production and Ca^2+^ uptake in mitochondria (Marchi et al., 2018), providing the bioenergetic basis for adaptation to ER stress. Meanwhile, the abnormality and dysfunction of MAMs can also induce ER stress and unfolded protein response (UPR). Therefore, mitochondria-ER cross-talks play a key role in response to ER stress and UPR.

X-box binding protein 1 (XBP1) is a key regulator of UPR or ER stress response in the mammalian cell (Glimcher, 2010). Specifically, accumulating evidence manifests that XBP1s play a potential regulatory role in the progression of several cardiovascular pathologies, including atherosclerosis (Zeng et al., 2009), myocardial ischemia (Thuerauf et al., 2006), and cardiac hypertrophy (Duan et al., 2015), which can eventually lead to heart failure. In recent studies, XBP1 has been shown to be SUMOylated by PIAS2 at two lysine residues (K276 and K297) located in the C-terminal transactivation domain, and the transcriptional activity of XBP1s toward UPR target genes could be dramatically increased upon the removal of XBP1 SUMOylation site (Chen and Qi, 2010; Uemura et al., 2013). Additionally, SENP1, as a specific de-SUMOylation protease for XBP1, could enhance the transcriptional activity of XBP1 and reduce cell apoptosis caused by ER stress (Jiang et al., 2012). Activating transcription factor 6 (ATF6), a crucial sensor of ER stress, is widely involved in the ER stress-mediated cardiomyocyte function in the heart (Jin et al., 2017; Blackwood et al., 2019). ATF6 can be modified by SUMO and its transcriptional activity is negatively regulated by SUMOylation, which is mediated by SUMO-1 and PIAS1 at Lys149, a conserved SUMOylation residue located at the N-terminus of the activated form of ATF6 (Hou et al., 2017).

**FIGURE 4:**
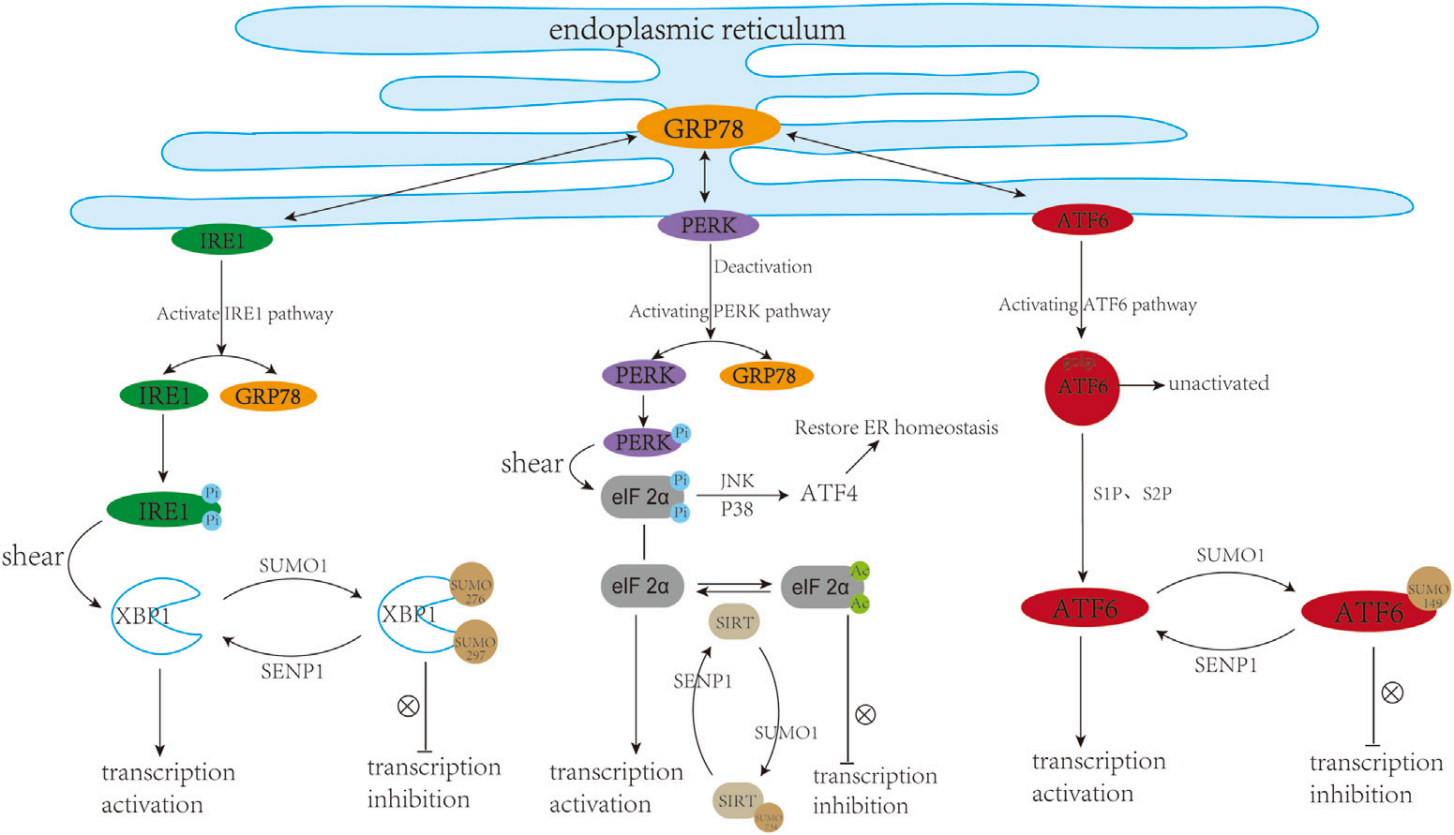
SUMO and cardiac endoplasmic reticulum stress. ER stress usually triggers three downstream pathways including IRE1, PERK, and ATF6 signalling pathways. In a resting state, IRE1, PERK, and ATF6 can bind to GRP78, while they are separated from GRP78 upon ER stress. (1) The dissociated IRE1 undergoes self-phosphorylation and P-IRE1, as an endonuclease, can cleave XBP1. XBP1 can translocate into the nucleus and trans-activate target genes. SUMOylation of XBP1 at K276 and K297 inhibited its transcriptional activity. (2) The dissociated PERK can undergo its own phosphorylation, followed by phosphorylation of eIF2 α. On the one hand, eIF2α can be phosphorylated and upregulate the level of ATF4 through JNK, P38 and other pathways, thereby restoring ER homeostasis. On the other hand, eIF2α can translocate into the nucleus and activate the transcription of target genes. Deacetylation modification of eIF2α at K143 induced by Sirt1 can protect cardiac cells from ER stress. SUMOylation of Sirt1 at K734 to Enhance its deacetylase activity. (3) During ER stress, ATF6 is cleaved into active ATF6 (cleaved-ATF6) under the action of S1P and S2P proteases. ATF6 can be SUMOylated at K149 and its transcriptional activity is negatively regulated by SUMOylation. This figure was created using Adobe illustrator cc 2018 (https://www.adobe.com/cn/products/illustrator.html).

Sirtuin 1 (SIRT1), an NAD + -dependent histone deacetylase, is widely present in organisms and plays a vital role in cardiac metabolism and ER stress. Inhibition or reduction of sirtuin-1 (SIRT1) activity can increase ER-induced myocardial damage, and STAC-3 (SIRT1 activator) has a protective effect on cardiomyocytes. Expression profile analysis of the three main UPR markers (PERK/eIF2α, ATF6 and IRE1) demonstrated that SIRT1 could prevent cardiomyocytes from ER pressure-induced apoptosis process by reducing the activation effect of the PERK/eIF2α pathway (Prola et al., 2017). SIRT1 deficiency leads to abnormal mitochondrial function in the adult heart, which in turn causes cardiac dilated cardiomyopathy (Planavila et al., 2012). (−)-Epicatechin (EPI) could inhibit cardiac fibrosis by enhancing the SUMO1 modification of SIRT1 and anti-fibrotic effect of SIRT1-SUMO1 could be mediated by the AKT/GSK3b signalling pathway (Luo et al., 2022). SUMOylation of SIRT at the C-terminal region (K734) can enhance the deacetylase activity of SIRT1 (Yang et al., 2007). Therefore, is it possible to improve the abnormal ER stress response by regulating the SUMO cascade reaction to change the SUMOylation state of SIRT1 (resulting in a similar effect to STAC-3) in the heart? This remains to be further elucidated. In summary, SUMO participates in ER stress from multiple levels, so it is necessary to find and identify the SUMOylation sites in ER stress-related proteins and test their effects in treatment of heart diseases. (Figure 4).

## 9 Conclusion and perspectives

Advances in the knowledge of the SUMO cascade system and the development of innovative tools are leading the way for the future application of SUMOylation in the cardiovascular system. In the past few years, the number of SUMO targets has been steadily increased, enabling more substrates to be included in the SUMOylation list in future. Notably, the efficacy of SUMOylation/deSUMOylation system in the heart is just emerging. Additionally, little is known about the specific function of SUMO1 and SUMO2/3 in the cardiovascular system. Similarly, the deconjugation effect of SENPs in specific cells is still unclear.

In the past, it was difficult to identity and analyze endogenous SUMO-protein complexes due to the low abundance of SUMOs, but now emerging mass spectrometry technology allows highly sensitive proteomic identification and SUMO modification site identification of SUMO targets in tissue cells, animals and human samples (Tammsalu et al., 2015; Hendriks and Vertegaal, 2016). This will help to define the SUMO conjugate proteome in heart tissues under the physiological and pathological environment. Furthermore, advances in single-cell sequencing technologies have a transformative impact on biomedical research, allowing the identification of rare subpopulations of cells, as well as the cellular-trajectory analysis of each cell’s transcriptome mediated by the SUMO system (Martini et al., 2019; Paik et al., 2020). Multimodal single-cell platforms can be applied to integrate and evaluate the SUMO system in cell population heterogeneity and its contributions to patient-specific drug responses and adverse effects.

Based on above-mentioned findings showing an association between SUMOylation and cardiovascular diseases, targeting the SUMO pathway for cardiovascular disease therapy is one promising approach. Multiple inhibitors and agonists targeting SUMO modifying enzymes have been explored and identified. An inhibitor of SUMO1 E1 enzymes, Ginkgolic acid, is reported to suppress myocardial infarction-induced cardiac fibrosis (Qiu et al., 2018). The another compound “N106” has been identified as an agonist of SUMO1 E1 enzymes and improve cardiac function in mice with heart failure by enhancing SUMOylation of SERCA2 (Kho et al., 2015). Compared to E1 and E2 enzymes, SUMO E3 ligase may be a more specific target for SUMO modification of substrates. SUMO deconjugating enzyme SENP family are also considered as effective targets for drug development. A flavonoid quercetin was found to inhibit SENPs and display protective effects in neuronal cells exposed to oxygen-glucose deprivation. Some studies have reported cardioprotective effects of quercetin against I/R injury (Lee et al., 2016). However, similar catalytic sites and protein structures are also major disadvantages in designing and developing selective SENP inhibitors, limiting the development of the novel therapeutic strategies towards SUMO-dysregulated cardiovascular diseases.

Among the compounds targeting SUMOylation, TAK-981 is currently the only compound that has been clinically tested in several types of tumor patients, such as head and neck cancer, lymphoma and myeloma [ClinicalTrials.gov Identifer: NCT03648372, NCT04065555, NCT04074330, NCT04381650 and NCT04776018]. TAK-981 can bind to the C-terminus of SUMO protein and the binding of TAK-981 to SUMO protein prevents the transfer of SUMO from E1 enzyme to E2 enzyme. A study on a novel SENP1 inhibitor senpPNA-R8 lays a certain foundation for subsequent clinical work [ClinicalTrials.gov Identifer: NCT03798587]. SenpPNA-R8 was found to inhibit the growth and metastasis of osteosarcoma (OS) under normoxic and hypoxic conditions. The main purpose of this early phase clinical study is to investigate its ability to inhibit SENP1 and penetrate into isolated human OS tissues *in vitro*.

For different heart failure patients, the types and degrees of heart failure, as well as the specificity of SUMOylation and the difference of SUMO target proteins, need to be considered in the clinical treatment. Therefore, individualized analyse and treatment may be an effective therapeutic way for different heart failure patients. However, the main challenge faced by this strategy is the global and precise identification of the multiple components participated in SUMOylation, including SUMO1/2/3, the various enzymes involved in this modification process and target proteins that undergo this modification. In fact, only a handful specific SUMOylated proteins have been identified in adult heart. Therefore, identifying more new endogenous SUMO targets and analyzing SUMOylation sites on factors is still very important to better understand the specific functions of SUMOylated proteins in the heart and to use them as new therapeutic targets in heart disease. In addition, the interaction between SUMO modification and other PTMs can make this issue more complex. In different situations, ubiquitination and SUMOylation can synergize or antagonize each other to regulate protein function. SUMOylation usually can increase protein stability through competitive binding to the same sites of ubiquitination. The interaction between protein ubiquitination and SUMOylation greatly enriches the complexity of the two PTM functions. Therefore, special attention should also be paid to the interactions and effect of these modifications in clinical therapeutics.

Collectively, the SUMO-mediated conjugation and deconjugation effects in the cardiovascular system are complex and many issues remain to be addressed. For example, how SUMO enzymes work in various cellular compartments and how the environment influences their behaviors remain unclear. Additionally, the expression of SUMO1 and SUMO2/3 and the binding abundance of the substrate under different conditions/stimuli (basal condition, ischemia, hypoxia, hypertrophy) need to be identified. Moreover, the differences in the expression and distribution of SUMOs and SENPs in different cell types of different species remain to be clarified. A better understanding of these issues contributes to elucidate the role of SUMO system in heart regulation and the development of novel therapeutic strategies for cardiovascular diseases.
